# Ethical challenges experienced by prehospital emergency personnel: a practice-based model of analysis

**DOI:** 10.1186/s12910-022-00821-9

**Published:** 2022-08-12

**Authors:** Henriette Bruun, Louise Milling, Søren Mikkelsen, Lotte Huniche

**Affiliations:** 1 The Prehospital Research Unit, Region of Southern Denmark, Odense University Hospital, Odense, Denmark; 2 The Mobile Emergency Care Unit, Department Anaesthesiology and Intensive Care Medicine, Odense University Hospital, Odense, Denmark; 3 Department of Psychology, The Faculty of Health Sciences, University of Southern Denmark, Odense, Denmark; 4 Psychiatric Department Middelfart, Mental Health Services in the Region of Southern Denmark, Middelfart, Denmark

**Keywords:** Ethics, Challenges, Emergency, Prehospital, Qualitative research

## Abstract

**Background:**

Ethical challenges constitute an inseparable part of daily decision-making processes in all areas of healthcare. In prehospital emergency medicine, decision-making commonly takes place in everyday life, under time pressure, with limited information about a patient and with few possibilities of consultation with colleagues. This paper explores the ethical challenges experienced by prehospital emergency personnel.

**Methods:**

The study was grounded in the tradition of action research related to interventions in health care. Ethical challenges were explored in three focus groups, each attended by emergency medical technicians, paramedics, and prehospital anaesthesiologists. The participants, 15 in total, were recruited through an internal information network of the emergency services. Focus groups were audio-recorded and transcribed verbatim.

**Results:**

The participants described ethical challenges arising when clinical guidelines, legal requirements, and clinicians’ professional and personal value systems conflicted and complicated decision-making processes. The challenges centred around treatment at the end of life, intoxicated and non-compliant patients, children as patients—and their guardians, and the collaboration with relatives in various capacities. Other challenges concerned guarding the safety of oneself, colleagues and bystanders, prioritising scarce resources, and staying loyal to colleagues with different value systems. Finally, challenges arose when summoned to situations where other professionals had failed to make a decision or take action when attending to patients whose legitimate needs were not met by the appropriate medical or social services, and when working alongside representatives of authorities with different roles, responsibilities and tasks.

**Conclusion:**

From the perspective of the prehospital emergency personnel, ethical challenges arise in three interrelated contexts: when caring for patients, in the prehospital emergency unit, and during external collaboration. Value conflicts may be identified within these contexts as well as across them. A proposed model of analysis integrating the above contexts can assist in shedding light on ethical challenges and value conflicts in other health care settings. The model emphasises that ethical challenges are experienced from a particular professional perspective, in the context of the task at hand, and in a particular, the organisational setting that includes work schedules, medical guidelines, legal requirements, as well as professional and personal value systems.

## Background

The bioethical literature traditionally focuses on overriding themes such as end-of-life care, consent, coercion, and priority setting. These themes commonly reflect serious ethical dilemmas marked by clear-cut value conflicts. In daily clinical practice, however, healthcare professionals are faced with medical, legal, and value-related considerations [1, 2] which are deeply entangled with specific priorities regarding particular patients and their relatives in diverse circumstances. Applying bioethics to the complexity of ethical challenges in clinical practice can be difficult. Further, Bringedal et al. [3] conclude that many situations perceived by doctors as entailing ethical dilemmas are rarely discussed in the bioethics literature. Similarly, Hem et al. conceptualise an ethical challenge in daily clinical practice as “a situation where there is doubt, uncertainty or disagreement about what is morally good or right” [4]. Ethical challenges have been researched empirically in different clinical practices like somatic medicine [5, 6], mental health [7–9], community health [10, 11], and among all types of healthcare professionals [12, 13]. Moreover, ethical challenges have been found to cause moral distress [13, 14]. As a result, various types of clinical ethics support services have been established to assist healthcare professionals in managing ethical challenges. Important examples are clinical ethics committees [15], clinical ethics consultation [16], moral case deliberation [17], and ethics reflection groups [18, 19]. A literature review by Haan et al. [20] shows the impact of moral case deliberation in healthcare settings. They find that moral case deliberation contributes positively to interprofessional interactions in promoting feelings of relief, relatedness and confidence, along with a better understanding of the perspectives of colleagues, one’s own perspective and the moral issue at stake. Moral case deliberation also contributes to an awareness of the moral dimensions of one’s own work and the importance of reflecting on them. Moreover, the literature review highlights how moral case deliberation can bring about changes in how patients and families are cared for and in how health care is organised. A small number of articles describe ethical challenges in emergency medicine [21–27], but little is known about how prehospital emergency personnel experience and manage ethical challenges. Emergency medicine constitutes a specialised clinical practise; for the most part, decision-making takes place in everyday life, under time pressure, with limited access to information about a patient and with few possibilities of consultation with colleagues. Even if the ethical challenges are in many respects similar to those clinicians face in other areas of health care, the circumstances in which the ethical challenges have to be managed are specific to emergency medicine. Further, prehospital emergency personnel spend most of their working hours “in the field”, which limits the practical establishment of workable forms of ethics support.

### Purpose of the research project

The results presented in this paper are part of a larger action research project conducted in collaboration with prehospital emergency personnel in the region of Southern Denmark. The overall purpose of the project is to develop and test an approach to clinical ethics support that is sensitive to the context of emergency medicine in local organisational settings. To inform the development and testing of a context-sensitive approach we explored the experiences with and the management of ethical challenges among prehospital emergency personnel. Knowledge of how ethical challenges are experienced and managed in clinical practice is important both for developing awareness and skills in support of the decision-making processes in complex and ethically challenging circumstances and for developing workable forms of ethics support. To support the purpose the research project is grounded in practice philosophy and empirical ethics, rather than in the broader philosophical tradition of applied ethics [28, 29].

In this paper, we report on ethical challenges experienced by prehospital emergency personnel. A further paper (in preparation) reports on how prehospital emergency personnel manage such challenges in their day-to-day work. Further, a context-sensitive approach to clinical ethics support will be developed and tested in collaboration with 3–4 local prehospital emergency units. Supporting and qualifying the management of ethical challenges is potentially beneficial not only to prehospital emergency personnel but to patients, relatives, external collaborators and the organisation of prehospital services.

## Method

### Emergency services in the Danish healthcare system

All Danish citizens have free access to healthcare. This includes the Danish prehospital emergency medical system which is a publicly funded, nationally implemented system providing the same basic services to all citizens in Denmark. The emergency medical system is a three-tiered system in which the basic unit is an ambulance staffed by two emergency medical technicians (EMT) or paramedics (PM) [30, 31]. The second tier consists of a PM in a rapid response vehicle while the third tier consists of a prehospital anaesthesiologist in either a ground-based mobile emergency care unit (MECU) or a helicopter [32]. The prehospital services are dispatched by an emergency medical dispatch centre in each of the five health regions in Denmark. Based on the information conveyed by the caller, the centre dispatches either an ambulance, an ambulance and a PM in a rapid response vehicle, or an ambulance and an anaesthesiologist-staffed MECU [33].

This investigation took place in the Region of Southern Denmark, one of Denmark’s five health regions, which covers an area of 12.191 km^2^ with a population of 1.2 million inhabitants. In this region, approximately one-quarter of the missions are combined missions in which both an ambulance and a MECU are dispatched. The other three-quarters of the missions are carried out by the ambulance crew alone [34]. An anaesthesiologist in a MECU serves as a medical backup for the ambulance crew.

### Research design

The overall methodological approach is founded on the tradition of action research related to interventions in health care [35, 36]. Action research offers a relevant research strategy when aiming to identify, develop and test clinical ethical support that is practice-based and sensitive to the organisational context of prehospital emergency medicine. Through the active involvement of prehospital emergency personnel, clinical ethics support can be developed and tested in this context in accordance with local workplace routines and culture. The action research approach thus supports the aim of aligning ethics support with the organisational, practical, and cultural aspects of clinical practice by involving the concerned parties. The core researcher group of this project consists of the authors of this paper. We involved the MECU anaesthesiologists, the head of quality, Ambulance Syd, the emergency medical system of the Region of Southern Denmark, the EMTs and PMs, prehospital educators, and a prehospital communications officer in ad hoc discussions of research activities, data analysis, communication, and the planning and testing of a context-sensitive approach to clinical ethics support.

### Data collection

The results presented in this paper are based on data from three semi-structured focus groups which were conducted to investigate how EMTs, PMs, and MECU physicians experience and manage ethical challenges in prehospital emergency medicine [37, 38]. We chose focus groups with several aims in mind. First, to gain insight into the broadest possible range of ethical challenges and management strategies relative to available time and resources for the investigation. Second, to gain insight into experiences, management strategies, reflections and contrasting views amongst colleagues. Third, to gain insight into the different perspectives of EMTs, PMs and MECU physicians. Lastly, to introduce the clinicians to the notion that ethical challenges are integral to any health professional practice and that such challenges can be systematically described and reflected on in collaboration with colleagues. The participants were recruited through an internal information network. In this local setting, most of the ambulance crews are formed by permanent pairs so that each PM or EMT teams up with the same partner during most shifts. The majority of the anaesthesiologists have worked at the MECU since its inception in 2006 and their prehospital workload on average amounts to two to five monthly 24-h shifts. One of 16 EMTs or PMs with supplemental training assists the MECU anaesthesiologists. These assistants drive the rapid response vehicle and assist in preparing medicine and performing additional duties. In total, 15 EMTs, PMs, and MECU physicians participated in the three focus groups. Table 1 shows the gender distribution and clinical background of the focus group participants. The nine EMTs and PMs had on average been employed in a prehospital service for 15.3 years, ranging from 6 to 28 years. The six MECU physicians had on average been employed in a prehospital service for 13.9 years, ranging from 4 to 30 years.

**Table 1:** Focus group participants

	Prehospital emergency personnel	
	MECU physicians	EMT and PM
Focus group 1	2 males, 0 female	2 males, 0 female
Focus group 2	1 male, 1 female	4 males, 0 female
Focus group 3	1 male, 1 female	2 males, 1 female
Total	4 males, 2 females	8 males, 1 female

Each focus group lasted two hours. The focus groups were conducted in a meeting room at the base station of the prehospital unit. Tables and chairs were arranged in a square, enabling all participants to face each other. As the focus groups took place during the late hours of the afternoon, sandwiches and soft drinks were provided. Authors HB and LH participated as facilitators in all three focus groups, while author LM participated as an observer. The facilitators and the observer were seated amongst the participants. One facilitator took notes on a paper flip-over board in plain view and for all to comment on. The observer managed the audio recordings and collected written consent forms. Author SM did not participate in the focus groups in any capacity due to his position as lead consultant of the MECU. The focus groups were guided by the following questions:

1. When and what kind of ethical challenges do you experience in your work?
2. How do you manage these ethical challenges?
3. In what ways does your workplace provide support for managing ethical challenges?

The flip-over charts were photographed and stored electronically. The focus group interviews were audio-recorded and transcribed verbatim. During transcription and further processing of data any names of persons and places mentioned during the focus groups were anonymised. Data was stored on a secure server.

### Data analysis

NVivo (QSR International, Burlington, Massachusetts, USA) was used to systematise data for analysis. The analytic procedure was based on systematic text condensation [39]. First, all transcripts were read to gain an overall impression of the content and a first impression of how the questions of the focus group guide had been addressed. Then, an iterative process was initiated, involving meaning condensation of each transcript followed by moving back and forth between the condensed meaning units and theoretical concepts. Accordingly, interactional aspects and content were integrated into the analysis. In the result section, text examples are presented without redundant utterances.

## Results

Overall, ethical challenges were experienced when clinical guidelines, legal requirements and clinicians’ professional and personal value systems conflicted with and complicated the decision-making processes. The prehospital emergency personnel experienced ethical challenges in three interrelated contexts: (1) when caring for patients, (2) being part of a prehospital emergency unit, and (3) during external collaboration.

### Ethical challenges in the context of caring for patients

When caring for patients, a frequent ethical challenge experienced by the prehospital emergency personnel was that patients’ autonomous decision-making capacity was reduced or even absent. For example, patients might be unconscious or semi-conscious due to cardiac arrest or severe trauma; their cognitive functioning might be impaired due to organ malfunctioning, head injury, anxiety, pain, shock and similar events. When patients´ autonomous decision-making capacity was reduced or absent, it was difficult and sometimes impossible for prehospital emergency personnel to honour patients’ rights to choose for themselves. This posed a challenge, as clinicians must fulfil their clinical duty to help the patient and determine the best course of action in a specific situation and for a specific patient. In the following, some examples of ethically challenging situations or questions concerning old patients at the end of life, intoxicated patients, children as patients, and relatives in various capacities are presented.

### Old patients at the end of life

In some situations, the prehospital personnel considered the treatment of old patients at the end of life to be doing more harm than good. Therefore, the best course of action would be regarded as abstaining from further interventions. In all three focus groups, EMTs and PMs discussed situations where they were summoned to old patients suffering from cardiac arrest, either in their private homes or in a nursing home. In situations without advance directives concerning resuscitation, a clinical evaluation discouraging any treatment could conflict with clinical guidelines and legal requirements. If the patient in question was frail and old, in a vegetative state and dependent on life support, or suffering from multiple comorbidities the best course of action might be no action at all. However, EMTs and PMs were legally required to start resuscitation and continue until a physician declared that further intervention was futile. In particular, experienced EMTs and PMs found it challenging to put legal requirements before what they considered to be in the best interest of a specific patient, for example for a patient not to be restored to a life of unconscious vegetation or debilitating illness, and to be granted a dignified death.

#### Text example 1: end of life

*Yes, we may hurry slowly, for example in cases where we are summoned to an elderly citizen with cardiac arrest. You can immediately tell that, well first and foremost the citizen is frail. We attach the electrodes and stuff and ascertain that the patient is asystolic—we do not know for how long. We can tell, well… the patient is dead, and our guidelines say we must continue with chest compression until a doctor arrives and says we can stop. So we contact the doctor as fast as we can and let them know that the patient is actually dead [and resuscitation should be stopped]. You see, we have been working as EMTs for so many years, I think we have a very clear idea of whether the patient has just died or has been dead for a while. So sometimes, to put it nicely, the chest compression is not delivered wholeheartedly.*

In this example patient autonomy was absent. Therefore, prehospital emergency personnel must draw on values other than patient autonomy. When resuscitation was no longer appropriate, EMTs and PMs focused on not doing harm and securing the dignity of the recently deceased patient. However, these may conflict with values implicit in clinical guidelines and legal requirements.

### Intoxicated patients

Sometimes patients’ autonomous decision-making capacity was reduced by alcohol or substance use.

#### Text example 2: intoxicated patients potentially at risk to themselves

*With intoxicated patients, it can sometimes be a little difficult, because on balance, ‘how alcohol-intoxicated is the individual? Do we think he is able to take care of himself, yes, no, why?’ Moreover, I would say, we have been faced with situations where he may be on the verge of being too drunk to take care of himself, but still not drunk enough to totally lack the ability to care for himself. Then you are faced with the fact that as soon as you say goodbye to them and leave them at the scene, they are on their way out the door, and we run the risk that they fall down the stairs. Therefore, you may just stand there with your arms crossed, well, if he falls, we must take it from there. If he suddenly knocks himself out, then that is a good reason to take him in [to a hospital].*

In cases of intoxicated patients falling down staircases or stumbling around in the streets, prehospital emergency personnel might conclude that medical examination in a hospital was important to treat injuries and monitor further developments. As seen in text example 2, prehospital emergency personnel might want to prevent harm and comply with their duty to help. This was attempted by establishing a trusting relationship with the patient. Nevertheless, intoxicated patients sometimes opposed treatment even though every measure was taken to convince them that medical assistance was warranted. In text example 2, prehospital emergency personnel struggled to evaluate whether the patient had values other than preserving health and respecting the patient's right to self-determination relative to the extent of their cognitive and autonomous decision-making abilities being affected by intoxication.

#### Text example 3: intoxicated in need of medical attention

*Someone who has just stumbled around in the street and does not want to go [to the hospital], and you think that swelling [on the forehead] should be checked by a doctor, and there is something about the patient being on blood thinners too. We are fully aware of this, but the patient has a different opinion. What to do? It is difficult, you would have to press the buttons and activate the psychiatrist [to be able to take the patient to a hospital], which means that I declare the patient insane and that’s not very nice. That could be taking a road away from ethics and creating a situation that, at least for the patient, would look like coercion.*

In text example 3, prehospital emergency personnel could not rely on the patient’s autonomy and ability to determine for himself. In the context of caring for the patient, opposing values were considered as doing good for the patient’s health and safety at the expense of doing harm by acting against his will. In the focus groups, prehospital emergency personnel conveyed that intoxicated patients were vulnerable but also difficult to help within the context of emergency medicine. In the context of emergency care, there was a risk of violating the value of providing equal and fair treatment to patients independent of their social status.

### Patients whose needs cannot be met by the emergency medical services

In some instances, a patient repeatedly reported convulsions or serious cardiac problems and called for emergency medical assistance. During the first couple of call-outs, the prehospital emergency personnel acted quickly and according to guidelines. Gradually, it became clear that there might be other underlying causes for the patient’s repeated contact with the prehospital emergency services, such as mental health issues or profound loneliness.

#### Text example 4: loneliness

*Part of the problem may be loneliness. There are some repeat callers who are not really sick, but the emergency medical system uses many resources on these people. Maybe they have a history where something has been ailing them in the past, and it has then elicited a certain response. They may have previously experienced an emergency response where a lot of nice people were gentle and caring. That kind of care is perhaps very welcome today when life hurts a little. We usually see through it after a few responses. Then the approach to the patient becomes different, and then the ethical dilemmas begin to stack up.*

In text example 4, prehospital emergency personnel tried to do good and form a treatment alliance with the patient. However, gradually they experienced a sense of futility, as they could not remedy the patients´ suffering or get them the help they might need. As emergency services are not supposed to allocate resources to these kinds of responses clinicians were left morally distressed. Moreover, they found their duty to help was undermined when collaborators outside emergency medicine, such as emergency psychiatric services, did not fulfil their obligation to help this group of patients.

### Children as patients

Children and adolescents under the age of 18 made up a particular category of patients. Depending on the age of the child and the severity of the situation, parents, guardians and other close relatives were implicated in different ways. In addition, working with children was emotionally demanding for most clinicians. Several examples of young children dying at home were mentioned during focus groups. Such events shifted the focus from caring for the patient to caring for the parents, siblings and others present at the scene, including colleagues. In the prehospital setting, no procedure was available to address the emotional needs of those present at a child´s death. Clinicians might be left to decide whether to help the bereaved by transporting the deceased child and close family members to the hospital for support, in some cases pretending to continue resuscitation and thereby tasking in-hospital colleagues with work that is furthermore emotionally taxing.

#### Text example 5: a child is dead

*The child was born two weeks before term, and this is on the day of term, and this child is… she is dead. And we work as hard as we can, and everything that can be done is being done, and we… the doctor decides that we continue [resuscitation] and take the child to the hospital because there are just many more automatic processes going on. We take the child to the hospital for the sake of the parents. Obviously, we cannot just say: ‘what a pity, call an undertaker, and our general practitioner will be here in six hours.’ That´s not an option. No, there has to be someone to take care of these parents.*

In text example 5, prehospital emergency personnel weighed up considerations for the dead child against the needs of the parents. They did so even though they violated clinical guidelines.

In other cases, it was ethically challenging when prehospital emergency personnel were summoned to children without any signs of severe disease but ended up taking the child to the hospital for further examination to calm anxious and insecure parents. It might not be in the best interest of the child to be taken to the hospital, nor might it be in the child’s best interest to be left with extremely worried parents. In some situations, prehospital personnel might suspect negligent care. This suspicion might arise when summoned to attend to a sick child or from observations made when attending to another member of the household. Despite their extended legal obligation to notify social services, prehospital emergency personnel found these situations ethically challenging. Unless there were obvious signs of neglect such as abuse or malnutrition, clinicians based a decision regarding notification of the social services on a mere snapshot of the child’s circumstances. Decision-making was complicated further by clinicians having no say in what happens next and no means of knowing whether notification would turn out to be helpful or might ultimately cause more harm than good to the child.

### Relatives in various capacities

When a patient’s autonomy was reduced, relatives or others who were involved in the life and care of a patient became important participants in the decision-making process. Ethical challenges could arise as prehospital emergency personnel took the considerations of relatives into account when evaluating the best interest of the patient. Relatives engaged in specific situations in very different capacities that could contribute positively to the decision-making process or might complicate matters. The relatives of a patient might constitute an invaluable source of information, for example about a patient’s medical history, cultural traditions and personal preferences. On the other hand, relatives might get in the way or even obstruct the efforts of prehospital emergency personnel, for example, if they are intoxicated, emotionally stressed or suffering from mental health issues. Relatives might insist on treatment and procedures that were not medically indicated, and might not be in the patient’s best interest. Some relatives might even have actively injured or caused harm to the patient. Finally, some relatives could be vulnerable and potentially in need of care themselves.

#### Text example 6: vulnerable relatives

*When should we just leave him to himself? We do not activate professional help, because he actually seems okay [after the sudden death of his life-long partner in their home]. This is at 11 pm. At four o´clock in the morning, we are dispatched to a traffic accident where I can [identify the patient] and say, I know him [from a previous call-out during this particular shift]. He took his car and then drove it as fast as he could, without wearing a seat belt, into one of the [concrete] blocks adjacent to the train bridge. I think we are often in situations in which, well what kind of information do I need to do my job, what do they [the relatives] want to hear, how should we tell, and how much care is needed afterwards? At some point, you have to leave the scene because we cannot stay and hold their hands for hours. So, I think it is difficult, when have you done enough and when can you leave?*

In text example 6, the prehospital emergency personnel were involved in a context of caring following the sudden death of an old woman. They established that nothing more could be done for her. The incident was devastating for her life-long partner who had been present during her death. Grief-stricken, his ability to take care of himself was seriously compromised. However, given the priorities of emergency medicine and working under time pressure, the prehospital emergency personnel could not cater for the needs of this relative.

### Patients with different value systems

Prehospital emergency personnel were confronted not just with patients and relatives, but also with patients’ social contexts and thus with different value systems. Participants in the focus groups explained how they were sometimes confronted with values grounded in cultural traditions that were unfamiliar to them. One example was a Muslim woman involved in a car accident. She wore clothes that covered her body except for her face and hands. To allow for a medical examination and possibly further action the PA and EMT removed the women’s clothes. By doing so, they were aware that they could be violating the women’s culturally based preferences while carrying out their duty to help. Moreover, they described situations where relatives or bystanders opposed their actions in more or less aggressive ways when proceeding in accordance with their duty to help, the clinical guidelines, and their legal obligations. In such situations, prehospital emergency personnel had to evaluate relatives’ or bystanders’ views, interests, and relationships with the patient. In addition, some bystanders might be distressed in ways that affected the prehospital emergency personnel's evaluation of what was in the patient’s best interest, such as carrying out a medical examination or protecting a woman’s dignity. These considerations were further complicated by the risk to personnel's safety while doing their job. If they did not examine Muslim women according to clinical guidelines, they might violate both their professional and personal values, as well as the value of providing equal and fair treatment to patients irrespective of gender or religion.

### Ethical challenges in the context of the prehospital emergency unit

Prehospital emergency personnel performed their work within an organisational context consisting of clinical guidelines, legal requirements, professional values [40, 41], and material and structural conditions. Some examples of ethically challenging situations or questions, which might arise here, were: Guarding the safety of oneself and colleagues; prioritising scarce resources; and staying loyal to colleagues who based their clinical decisions on different values than their own.

### Guarding safety

In some situations, the prehospital emergency personnel had to balance their duty to help a patient while safeguarding others and themselves. Some focus group participants described how transportation of very sick children often took place at high speed involving a threat to the safety of others and the clinicians themselves. Some participants talked about “professional tunnel vision” where they focus exclusively on their duty to help a patient who depends on them. In other situations, relatives might pose a threat to the prehospital emergency personnel while they were treating a patient. An example was a prehospital anaesthesiologist struggling to save a woman who suffered from severe bleeding while being threatened by her partner who appeared to be a gang member; “if she dies, you´ll die too”. In some situations, prehospital emergency personnel were threatened by relatives in ways that prevented them from acting in accordance with their duty to help a patient and they might have to call for police assistance.

#### Text example 7: relatives posing a threat to clinicians

*We arrive to find an unconscious woman, lying in bed and we are in no doubt that she needs to be taken to the hospital. Then her husband enters the room. He is wearing all sorts of chains and has piercings in his ears and eyebrows, and he starts to fumble with a lot of medicine in this bedroom. Now, we are three people in one ambulance and two [EMTs] in another ambulance, and we all agree that she's “critical” [in a very critical condition]. Then the doctor asks the husband what diseases she has, and then suddenly, he explodes and says he's told them that damn thing already once… is [the doctor] stupid or what? It all ends with the husband threatening to kill us if we touch his wife.*

In text example 7, prehospital emergency personnel were not in doubt about how to do good and how to prevent harm to the patient. She urgently needed hospital treatment. However, within the context of emergency care, they also had to guard their own safety. It was distressing both to be subject to threats and to postpone their duty to help until the police arrived.

### Prioritising time

Many focus group participants reflected on how prehospital resources might be used responsibly and in the best interest of patient care. They acknowledged that the emergency medical dispatch centre sometimes prioritised assignments based on very little information and that the situation might be perceived to be a lot more serious by the emergency medical dispatch centre than it turns out to be on on-site evaluation. Even though EMTs and PMs found it difficult, they often considered whether they should try to persuade patients not to use the ambulance services, for example, if the patient had no clinically indicated need for emergency transportation to a hospital. These considerations were grounded in concerns to ensure sufficient capacity in case of other urgent events.

#### Text example 8: a patient with a broken finger occupies the ambulance

*Right now, I have to spend an hour and a half taking this patient, who in principle should drive himself, to the hospital. This means the ambulance will be occupied for an hour and a half and is taken out of active service just to transport a patient with a broken finger when in reality [the ambulance] should be used for something else. We do not know if in twenty minutes there will be a huge traffic accident out on the Great Belt Bridge. Well, we do not know. But one just tends to take them to the hospital anyway because it is the easiest solution.*

In text example 8, the prehospital emergency personnel tried to protect themselves and their specialized function against misuse of medical services provided by the welfare state. They evaluated the cost of use of their medical expertise and the potential benefits of its alternative use.

### Loyalty to colleagues

The prehospital personnel were expected to be loyal to a hierarchical structure and command, where physicians have the final say in the decision-making process. Some participants described value conflicts related to this hierarchical structure. In some situations, there might be a conflict of values between supporting a colleague and acting in solidarity with the patient.

#### Text example 9: colleagues put themselves first

*Sometimes, it is not about the patient, it is about it being night-time and you would like to get back to base and sleep. We may have had many call-outs, and then the right thing might have been to take the patient to the hospital to be examined. ‘What is the cause of these chest pains, are they chest pains or are they just…[something else].’ There may be some colleagues who actually misinterpret the situation and put themselves first, and then you can stand next to them and feel that you yourself would probably have done something else, but what is the threshold for intervening and objecting very loudly?*

In the text example 9, a prehospital emergency clinician disagreed with a more senior colleague. He found that his duty to help the patient collided with his duty to respect the hierarchical structures and the line of command in the organisation.

### Ethical challenges in the context of external collaboration

Frequently, decision-making took place in everyday life settings and might involve bystanders. Also, prehospital emergency personnel sometimes interacted or collaborated with a wide range of professionals representing other state institutions. These might include emergency departments at other hospitals, local municipal institutions such as nursing homes and shelters for the homeless, along with police authorities and social services. Examples of ethically challenging situations or questions were: Being summoned when others made decisions or took action; attending to patients whose legitimate needs were not met by the appropriate medical or social services; collaboration around end-of-life decisions; and working alongside authorities with different roles, responsibilities, and tasks.

### Ethical challenges related to collaboration with hospital departments

The participants in the focus groups described ethical challenges related to working with the staff at the hospital departments. Reflecting on a specific incident involving an emergency department, an EMT concludes:”…sometimes we are used as garbage collectors, for cleaning up when others may not be able to make decisions.” The EMT explained how the specialised neonatal ambulance was summoned to transport a newborn baby from a local hospital to a university hospital. While driving to the local hospital the ambulance crew was informed that the baby had been treated for cardiac arrest. As the ambulance arrived at the local hospital and the crew got ready to take over the responsibility for the treatment of the baby, it again had cardiac arrest. The crew then waited for several hours until the baby was stable. Once again, they prepared for transport, but as the baby was moved from the trauma unit a third cardiac arrest occurred. The local team worked intensely to save the child’s life. The EMT described a value conflict between loyalty towards a decision made by the local physician and his own evaluation of the situation as it had developed. At this stage, he considered that transportation of the baby for a long distance was not feasible due to the multiple cardiac arrests the baby had suffered over the past few hours. The ambulance personnel took over the cardiopulmonary resuscitation and the local team was urged to step aside with the physician operating the neonatal ambulance to reconsider the decision for transportation. This intervention paused the process and made way for a re-evaluation of whether transportation was indeed feasible and in the best interest of the patient and the relatives.

Prehospital emergency personnel also experienced ethical challenges when interacting with psychiatric emergency departments. In all focus groups, participants mentioned being regularly summoned to young women who displayed self-harming behaviour, commonly by cutting themselves, and how psychiatric emergency departments usually were involved. However, these young women were seldom psychotic or perceived as posing a real threat to themselves or others. Therefore, there were no legal grounds for retaining them against their will and they were generally discharged from psychiatric wards the day after admission. The same patient frequently called the emergency services for help within days and prehospital emergency personnel became involved again. They explained how their obligation to help was being challenged when the psychiatric departments did not offer treatment, which might make a real difference in these young women’s lives. Consequently, such call-outs took time and resources away from the core tasks of the emergency services.

### Local municipal institutions

The prehospital emergency personnel also collaborated with local municipal institutions such as nursing homes, homeless shelters, and treatment facilities related to alcohol and substance dependency.

The participants in the focus groups brought up ethical challenges related to collaboration around end-of-life decisions when treating patients in nursing homes. The nursing home staff on evening and night shifts were very often temporarily employed care assistants who might not know the patients very well and had no or limited medical training. They might call on the emergency services in situations when medical interventions were not necessarily in the best interest of old, fragile residents with comorbidities. The focus group participants discussed whether life should be preserved at all costs. Most agreed that dying with dignity in a nursing home was preferable to dying while invasive emergency medical interventions were carried out or dying while being rushed to a hospital. Prehospital emergency personnel perceived that most temporarily employed care assistants did not seem to recognise the value of a dignified death. Furthermore, care assistants usually did not know whether an advance directive existed, and when it did exist, they might not know where it was kept or they did not have access to it. Such circumstances challenged the collaboration. However, advance directives did not always solve the ethical challenges of securing a dignified death. Often, advance directives were not completed correctly or might be outdated. In these cases, EMTs and PMs were legally obliged to wait for a physician to decide on the termination of treatment.

#### Text example 10: the resuscitation papers are the work of the devil

*Then we arrive [at the nursing home] and find this person lying there and we are also told that there are papers stating that he does not want resuscitation. These papers are simply the work of the devil because there is always something wrong with them. There are a lot of requirements and criteria for the papers, and they are almost never met. And in reality, we are obliged to start treating the patient, but at the same time, we also know that it is somewhat an abuse of the patient in a situation where the whole family is present to watch.*

In text example 10, prehospital emergency personnel described a complex situation demanding a wide range of considerations. First, they wanted to respect the patient’s right to self-determination declared in the advanced directive, which was outdated and thus legally invalid. This challenged the prehospital emergency personnel’s respect for other health care professionals and their areas of responsibility. To honour the rights of the patient and to respect and do good for the relatives the prehospital emergency personnel decided to tolerate the violation of the legal requirement concerning up-to-date advanced directives.

Several participants in the focus groups described patients who discontinued their municipal alcohol treatment courses. In such cases, prehospital emergency personnel struggled to help the patient and found it difficult to leave them on their own, perhaps even without a place for them to stay overnight. The participants discussed the scope of their responsibility within the emergency services, and whether and how patients with a lifelong history of abuse really do benefit from the prehospital emergency interventions or the hospital treatments.

#### Text example 11

*It could also be a drug addict. Some of these people have lived a miserable life where you could say, ‘well now they have spent 50 years trying to ruin their health and finally they are succeeding. Should it be us and the system prolonging their misery further, or should we just provide some relief?’ It ought to be a matter for the general practitioner.*

In text example 11, prehospital emergency personnel wanted to safeguard emergency medical services and competencies for patients who required immediate medical attention. However, when personnel were confronted with patients who obviously had not profited from, for example, municipal alcohol treatment, they struggled with their duty to respect external collaborators, in light of their professional assessment and limits of their areas of responsibility and expertise.

### Police authorities

Ethically challenging situations could arise in relation to collaboration with police authorities, for example, when prehospital services were summoned in cases where a person had died unexpectedly or while being alone. In these cases, the police were required to investigate the death. Consequently, healthcare professionals and police officers attended for different reasons and had different tasks. Clinicians were tasked with confirming death and attending to the body while the police were tasked with assessing whether death occurred due to natural or illegal causes. These roles and tasks resulted in different approaches to the situation, the deceased, and any relatives present at the scene.

#### Text example 12: apologising on behalf of the police

*And then it gets out of hand, and you almost have to apologize to the relatives who show up at some point. Well, the police have to [investigate a sudden death]. [Even in a] very old little lady. No one could have any interest in killing her.*

Text example 12 was an example of colliding tasks and areas of responsibility between the police investigating a potential crime and the prehospital emergency personnel’s work within the remit of their duty to help patients and relatives. When the police were involved, it was a challenge for prehospital emergency personnel to witness and respect the way the police performed their job.

## Discussion

Our first point of discussion concerns the existing literature and how our study adds to this body of knowledge. When prehospital emergency personnel presented ethical challenges during the focus groups there was little mention of concepts and principles in moral philosophy or bioethics. Rather, participants described how they balanced responsibilities and values in each case. Some of these values reflected principlism (respecting patient autonomy, doing good, preventing harm and just allocation of resources), others pointed to deontology (acting under clinical guidelines and the legal requirements), and some values resembled consequentialism (evaluating the cost and potential benefit of alternative uses of the medical resources). Other values were akin to those espoused by an ethics of care (forming a treatment alliance with patients as individuals, acting in ways that support cooperation with the patient) [42]. There is limited literature reflecting the participants’ practice-based and contextual perspectives. Bringedal et al. [3] support this position by pointing out that bioethical debates have focused on a limited set of topics with classical and highly visible dilemmas. In addition, Bringedal et al. point to situations perceived by doctors as ethical dilemmas such as “prescription of addictive drugs or antibiotics on request; social contact with, and accepting gifts from patients; providing a strategic diagnosis for sick-leave certificates; withholding information about less important adverse events; the practice of “defensive medicine” with a low threshold for specialist referral”. The authors conclude that this “ethics of the ordinary” is more frequent, but less discussed and “these “ordinary” ethical dilemmas can be perceived as more challenging because there is less consensus and professional guidance on how to manage them”. In the context of emergency medicine a paper by Sandman aimed to “gather details of a wide range of ethical conflicts without, however, detailing the circumstances under which they occurred or whether carers solved the dilemmas differently under these circumstances” [25]. The results were presented under nine headings: (1) Patient’s best interest, (2) Patient’s self-determination, (3) Professional ideals, (4) Professional role and self-identity, (5) Organisational structure and resource management, (6) Social ideals, (7) Significant others and/or bystanders, (8) Other care professionals, (9) Other professionals (mainly police and firefighters).

In the context of caring for patients in emergency medicine, most studies on ethical challenges are focused on decision-making concerning cardiac arrest [22, 27, 43]. In the Region of Southern Denmark, cardiac arrest only accounts for 2.8% of the total number of emergency call-outs with light and sirens per year. Our study shows that although cardiac arrest comes with serious ethical challenges, so do other incidents, for example, acute intoxication, self-harm, and loneliness. Ethical challenges are also experienced in relation to patient autonomy, children as patients, different cultural traditions and values, and working under the threat of physical violence or verbal abuse.

To our knowledge, only one study addresses the issue of conflicting values between emergency personnel and emergency care managers [44]. Based on self-administered questionnaires and semi-structured interviews French et al. found that ethical conflicts between ambulance crew and their organisation principally arise from differences in their reasoning based on either rights or utility. In our study ethical challenges arise in the context of the prehospital emergency unit; guarding the safety of oneself and colleagues, prioritising resources, and staying loyal to colleagues who ground their clinical decisions in different values. Differences in how ethical conflicts arise between prehospital emergency personnel and their organisation would seem to arise due to how emergency services are embedded in wider structures of health care provision and funding.

Besides the study mentioned above by Sandman et al. [25] moral challenges of collaborating with relevant actors outside emergency medicine have not been investigated. In our study, the context of external collaboration holds important ethical challenges. Although prehospital emergency personnel often appear to share a professional value system, it is important to stress that ethical challenges also arise between EMTs, PMs, and anaesthesiologists, for example, related to differences in medical backgrounds, positions, responsibilities, tasks, professional values, and personal values. Jimenez-Herrera et al. [5] also report this. The study maps how nurses in emergency care experience ethical conflicts with other medical professionals in four areas: “autonomy”, “reification of the injured body”, “pain”, and “death”. Regarding conflicts around “reification of the injured body”, the study describes how nurses’ human-centred and holistic model of medicine challenges physicians’ technocratic model which entails a mind–body distinction and a conceptualisation of the body as a machine.

Our second point of discussion concerns the lack of conceptual systematisation in the analysis of ethical challenges encountered in emergency medicine. Some analyses of ethical challenges are characterised by a seemingly haphazard mixing of bioethical concepts and principles, professional and personal values, disease categories, clinical procedures, organisational conditions, and practical circumstances. A case in point is a study by Becker et al. [24] in which seven problem areas were identified and coupled with advice on how to manage them: (1) Delay or denial of transport for non-emergency conditions, (2) The use of light and sirens for patient transport, (3) Termination of resuscitation and medical futility, (4) Duty hours and maintenance of a competent emergency medical service workforce, (5) Substance abuse by emergency medical service providers, (6) Disaster management and triage, and (7) Maltreatment of children and the subsequent reporting of issues to the authorities [24]. Another example is the study of Torabi et al. [26] which aimed to describe the experiences of ethical challenges by prehospital personnel as divided into three categories: (1) Respecting client values, (2) Performing tasks professionally, and (3) Personal characteristics [26]. A literature review by Cheraghi et al. further highlighted the lack of conceptual distinctions in analyses of emergency medical technicians´ ethical challenges in prehospital emergency services as expressed in the following list of ethical challenges: “Decision over dispatching an ambulance to the scene, cardiopulmonary resuscitation, triage in crisis and disaster, irrational interventions of the patient or patient´s family, intervention per the patient´s informed consent, and safe driving” [45]. Some of these issues refer to decision-making, some to specific treatment procedures; others refer to prioritisation and circumstances at the scene; others refer to patients’ rights, and some indicate questions of safety. The lack of conceptual distinctions is unhelpful if the aim is to identify key aspects of how ethical challenges and conflicting values are part of emergency medicine and decision-making in specific circumstances within wider structures of the provision of health care and other welfare state services.

**Fig. 1:**
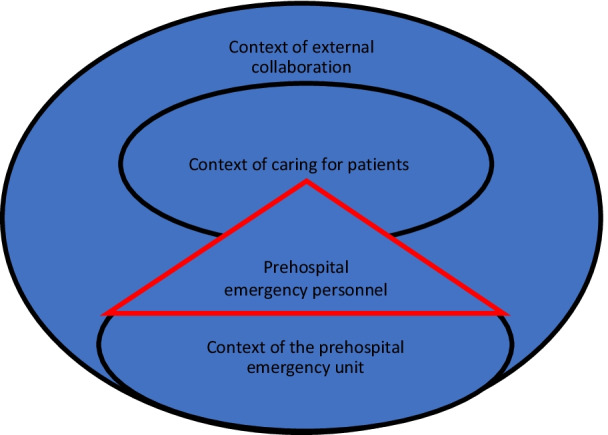
A practice-based model of ethical challenges and value conflicts

To address the lack of conceptual distinctions we suggest a model that builds on our practice-based and context-sensitive analysis of ethical challenges and value conflicts in emergency medicine (Fig. 1).

The practice-based and context-sensitive approach to our analysis of ethical challenges resulted in conceptual distinctions which assist the understanding of value conflicts in clinical practice as multi-layered and interwoven. Taking the professional perspective as a point of departure, we distinguished between different relevant contexts pertaining to the specific circumstances of caring for patients in emergency medicine. During the analytic process, it was established that prehospital emergency personnel experience ethical challenges as constituted in three interrelated contexts: when caring for patients, in the prehospital emergency unit, and during external collaboration. Most ethical challenges are experienced in the context of caring for patients, but ethical challenges experienced in the context of emergency medicine and relate to external collaboration also have a bearing on patient care. When ethical challenges arise, they commonly involve value conflicts pertaining to the provision of care that is in the best interest of the patient and their relatives, to the clinical guidelines and the legislation in the area of emergency medicine, as well as to interprofessional and personal value conflicts. In addition, broader cultural and societal value conflicts may come into place. At times specific persons embody value systems, at other times value systems implicitly frame the work of prehospital emergency personnel. This is the case when they evaluate a situation while taking into account a patient´s medical condition, the contribution of relatives, along with practical, organizational, and legal frameworks for acting in a patient’s best interest. In this way, we identified how each context in each particular situation of patient care is endowed with a particular set of value conflicts. Value conflicts arise within as well as across these contexts and must be considered jointly. Thus, ethical challenges arise in complex circumstances from the perspective of prehospital emergency personnel. Clinicians navigate and balance values concerning the care for a patient and others involved within the context of emergency medicine and in collaboration with colleagues and often also external professionals in public settings or private homes. Though emergency medicine is a specialised medical practice, the contextual analysis of how ethical challenges are experienced from the perspective of clinicians can be generalised to other areas of health care. In Table 2, we have listed some examples from the analysis of values and considerations that typically may come into conflict.

**Table 2:** Values and considerations that may come into conflict

Values in the context of caring for patients
Acting in accordance with the duty to help, clinical guidelines and legal requirements Forming a treatment alliance with patients as individuals Providing equal and fair treatment to patients, irrespective of age, gender, religion, and social status Respecting hierarchical structures and the line of command of the organisation Guarding the safety of patient, self, colleagues and others involved Evaluating potential cost and benefit of alternative uses of medical expertise
*Values in the context of external collaboration*
Acting in accordance with the duty to help, clinical guidelines and legal requirements Forming a treatment alliance with patients as individuals Providing equal and fair treatment to patients, irrespective of age, gender, religion, and social status Respecting hierarchical structures and the line of command of the organisation Guarding the safety of patient, self, colleagues and others involved Evaluating potential cost and benefit of alternative uses of medical expertise
*Values in the context of external collaboration*
Respect for non-prehospital healthcare professionals and external collaborators, including their professional assessment of the situation, their tasks and areas of responsibility Preventing harm and doing good for bystanders Respecting bystander views and needs, in light of their cognitive abilities, their intents and relationship to a patient
*Values pertaining to prehospital emergency personnel*
Acting in accordance with the value system of their specific health profession (EMT, PM, physician) Acting in accordance with their personal value system

## Conclusion

In this paper, we report on a study of ethical challenges in prehospital emergency medicine in the Region of Southern Denmark. Based on our study we present a model for analysis of how to distinguish between key ethical challenges and conflicting values in prehospital emergency work. At the core of this model is an understanding of ethical challenges as they arise from the perspective of clinicians and are constituted within three interrelated contexts: when caring for patients, being part of the prehospital emergency unit, and during external collaboration. The model offers an overall analytic framework which recognises ethical challenges as situated, relational, contextual, multi-layered and interwoven. The model offers the possibility of analysing how professional, personal, organisational, and broader socio-cultural value conflicts intersect and thus offers a way to qualify decision-making in ethically challenging circumstances.

## Data Availability

Individuals may be identified from audio files. In adherence to the regulations of the Danish Data Protection Agency, these are thus not available for public distribution. The anonymised transcriptions of audio files (in Danish) are available from the corresponding author on reasonable request.

## Abbreviations

EMT: Emergency medical technician
MECU: Mobile emergency care unit
PM: Paramedic
