# Author Correction: Ethical challenges experienced by prehospital emergency personnel: a practice-based model of analysis

**DOI:** 10.1186/s12910-022-00862-0

**Published:** 2022-11-26

**Authors:** Henriette Bruun, Louise Milling, Søren Mikkelsen, Lotte Huniche

**Affiliations:** 1 The Prehospital Research Unit, Region of Southern Denmark, Odense University Hospital, Odense, Denmark; 2 The Mobile Emergency Care Unit, Department Anaesthesiology and Intensive Care Medicine, Odense University Hospital, Odense, Denmark; 3 Department of Psychology, The Faculty of Health Sciences, University of Southern Denmark, Odense, Denmark; 4 Psychiatric Department Middelfart, Mental Health Services in the Region of Southern Denmark, Middelfart, Denmark

## Author Correction: BMC Medical Ethics (2022) 23:80 10.1186/s12910-022-00821-9

Following publication of the original article [1], the author has changed the table 2 and it should read as follows:

**Table:** 

**Values in the context of caring for patients**
Preventing harm and doing good for patients and relatives Respect for patient autonomy and right to self-determination in consideration of the cognitive ability of the patient Respect for the contributions and needs of relatives in consideration of their cognitive abilities, their intents, and relation to the patient
**Values in the context of the prehospital emergency unit**
Acting in accordance with the duty to help, clinical guidelines and legal requirements Forming a treatment alliance with patients as individuals Providing equal and fair treatment to patients, irrespective of age, gender, religion, and social status Respecting hierarchical structures and the line of command of the organisation Guarding the safety of patient, self, colleagues and others involved Evaluating potential cost and benefit of alternative uses of medical expertise
**Values in the context of external collaboration**
Respect for non-prehospital healthcare professionals and external collaborators, including their professional assessment of the situation, their tasks and areas of responsibility Preventing harm and doing good for bystanders Respecting bystander views and needs, in light of their cognitive abilities, their intents and relationship to a patient
**Values pertaining to prehospital emergency personnel**
Acting in accordance with the value system of their specific health profession (EMT, PM, physician) Acting in accordance with their personal value system

The original article has been corrected.

## References

[1] Bruun H, Milling L, Mikkelsen S (2022). Ethical challenges experienced by prehospital emergency personnel: a practice-based model of analysis. BMC Med Ethics.

